# Correlations of *MTHFR* 677C>T Polymorphism with Cardiovascular Disease in Patients with End-Stage Renal Disease: A Meta-Analysis

**DOI:** 10.1371/journal.pone.0102323

**Published:** 2014-07-22

**Authors:** Xian-Hui Gao, Guo-Yi Zhang, Ying Wang, Hui-Ying Zhang

**Affiliations:** 1 Laboratory of Preventive Medicine, School of Public Health, Liaoning Medical University, Jinzhou, China; 2 Department of Toxicology, School of Public Health, Liaoning Medical University, Jinzhou, China; 3 Sleep Monitoring Center, First Affiliated Hospital of Liaoning Medical University, Jinzhou, China; University of Florida, United States of America

## Abstract

**Objective:**

This meta-analysis was conducted to evaluate the correlations of a common polymorphism (677C>T) in the methylenetetrahydrofolate reductase (*MTHFR*) gene with risk of cardiovascular disease (CVD) in patients with end-stage renal disease (ESRD).

**Method:**

The following electronic databases were searched without language restrictions: Web of Science (1945∼2013), the Cochrane Library Database (Issue 12, 2013), MEDLINE (1966∼2013), EMBASE (1980∼2013), CINAHL (1982∼2013) and the Chinese Biomedical Database (CBM) (1982∼2013). Meta-analysis was performed using STATA statistical software. Odds ratios (ORs) with their 95% confidence intervals (95%CIs) were calculated.

**Results:**

Eight cohort studies met all inclusion criteria and were included in this meta-analysis. A total of 2,292 ESRD patients with CVD were involved in this meta-analysis. Our meta-analysis results revealed that the *MTHFR* 677C>T polymorphism might increase the risk of CVD in ESRD patients (TT vs. CC: OR = 2.75, 95%CI = 1.35∼5.59, *P* = 0.005; CT+TT vs. CC: OR = 1.39, 95%CI = 1.09∼1.78, *P* = 0.008; TT vs. CC+CT: OR = 2.52, 95%CI = 1.25∼5.09, *P* = 0.010; respectively). Further subgroup analysis by ethnicity suggested that the *MTHFR* 677C>T polymorphism was associated with an elevated risk for CVD in ESRD patients among Asians (TT vs. CC: OR = 3.38, 95%CI = 1.11∼10.28, *P* = 0.032; CT+TT vs. CC: OR = 1.44, 95%CI = 1.05∼1.97, *P* = 0.022; TT vs. CC+CT: OR = 3.15, 95%CI = 1.02∼9.72, *P* = 0.046; respectively), but not among Africans or Caucasians (all *P*>0.05).

**Conclusion:**

Our findings indicate that the *MTHFR* 677C>T polymorphism may be associated with an elevated risk for CVD in ESRD patients, especially among Asians.

## Introduction

Hemodialysis (HD) is a common form of dialysis therapy for kidney disease by achieving the extracorporeal removal of waste products such as creatinine, urea and free water from the blood [1]. HD is the most common procedure performed for patients with end-stage renal disease (ESRD), with an estimation of 1.5 million ESRD patients receiving HD worldwide [2]. Despite its important role in treating ESRD, HD also serves as a multiplier for other vascular risk factors, contributing to an increased incidence of cardiovascular disease, such as atrial fibrillation and myocardial ischemia [3], [4]. Moreover, it has been reported that mortality caused by cardiovascular disease (CVD) in ESRD patients is 25% higher than that in the general population [5], [6]. Therefore, the assessment and prevention of CVD are major considerations for dealing patients with HD [7]. Generally, CVD refers to any disease that affects the cardiovascular system, principally cardiac disease, vascular diseases of the brain and kidney, and peripheral arterial disease [8]. Clinically, traditional CVD risk factors include old age, male sex, diabetes mellitus, and systolic hypertension and so on [9]. Over past few decades, studies have demonstrated that the severity and extent of cardiovascular complications in ESRD patients is disproportionate to the number and severity of traditional risk factors [10], [11]. In recent years, nontraditional risk factors such as hemoglobin levels, inflammation, oxidative stress, and particularly epigenetic mechanisms of gene regulation have been emphasized as factors that may play an important role in the development of cardiovascular disease in patients with HD [12]. In addition, genetic polymorphisms have received considerable attention as a competitive candidate for the risk of CVD in ESRD patients [13].

Methylene tetrahydrofolate reductase (MTHFR), encoded by the *MTHFR* gene, is a key enzyme in folate metabolism which plays a critical role in the synthesis and methylation of DNA encoding for an enzyme responsible for re-methylation of homocysteine (Hcy) to methionine [14]–[16]. Actually, Hazra et al. confirmed that the functional polymorphism in MTHFR Ala222Val (also reported as MTHFR C677T), rs1801133, on chromosome 1 was associated with plasma homocysteine in a healthy population [17]. The human *MTHFR* gene is located on the short arm of chromosome 1 (1p36.3), is comprised of 11 exons and 10 introns, and is 19.3 kb in length [17]. Currently, multiple researchers have demonstrated that genetic variations in the *MTHFR* gene might alter its production and function, thereby affecting individual prevalence rates of some diseases and even cancers [18], [19]. More specifically, multiple polymorphic sites such as 677C>T (rs1801133) and 1298A>C (rs1801131) within the *MTHFR* promoter region have been reported in previous studies, of which 677C>T has been suggested to be implicated in the susceptibility to CVD in ESRD patients [20], [21]. It should be noted that plasma Hcy levels have been found to be an important risk factor for the development of CVD in ESRD patients [22]. However, polymorphism at nucleotide 677C>T may reduce the activation and thermo-stability of MTHFR, and subsequently inhibit the generation of 5-Methylenetetrahydrofolate and interfere with the normal metabolism and re-methylation of Hcy, thereby resulting in the accumulation of Hcy and an elevated plasma Hcy concentration in individuals; however, contradictory results have also been reported [23], [24]. Given the conflicting evidence on this issue, we conducted a meta-analysis of all available cohort studies to determine whether *MTHFR* genetic polymorphisms contribute to the pathogenesis of CVD in ESRD patients.

## Methods

### Publication search

The following electronic databases were searched without language restrictions: Web of Science (1945∼2013), the Cochrane Library Database (Issue 12, 2013), MEDLINE (1966∼2013), EMBASE (1980∼2013), CINAHL (1982∼2013) and the Chinese Biomedical Database (CBM) (1982∼2013). We used the following keywords and MeSH terms in conjunction with a highly sensitive search strategy: [“SNP” or “mutation” or “genetic polymorphism” or “variation” or “polymorphism” or “single nucleotide polymorphism” or “variant”] and [“cardiovascular disease” or “CVD” or “cardiovascular risk” or “Hemodialysis” or “HD”] and [“methylenetetrahydrofolate reductase” or “MTHFR” or “methylene tetrahydrofolate reductase” or “5,10-methylenetetrahydrofolate reductase”]. We also conducted a manual search to find other potential articles based on references identified in retrieved articles.

### Inclusion and exclusion criteria

The following criteria were used to determine the eligibility of included studies: (1) the study design must be a clinical cohort study; (2) the study must relate to the relationships of *MTHFR* 677C>T polymorphisms with the risk of CVD in ESRD patients; (3) all patients met the diagnostic criteria for HD; (4) the study must provide sufficient information about the frequencies of the *MTHFR* 677C>T polymorphism. If a study could not meet the inclusion criteria, it was excluded. The most recent or the largest sample size publication was included when the authors published several studies using the same subjects. The supporting PRISMA checklist is available as Checklist S1.

### Data extraction

Data were systematically extracted by two authors from each included study using a standardized form. The form used for data extraction documented the most relevant items including publication year, language of publication, the first author's surname, geographical location, sample size, design of study, the source of the subjects, source of samples, allele frequencies, genotyping method of SNP, evidence of HWE in healthy controls, etc.

### Methodological assessment

Methodological quality was independently assessed by two observers according to the Newcastle-Ottawa Scale (NOS) criteria [25]. The NOS criteria includes three aspects: (1) subject selection: 0∼4 points; (2) comparability of subject: 0∼2 points; (3) clinical outcome: 0∼3 points. NOS scores range from 0 to 9 with a score ≥7 indicating good quality. The supporting NOS score criteria are available in Supplement S1.

### Statistical analysis

Meta-analysis was performed with the use of the STATA statistical software (Version 12.0, Stata Corporation, College Station, TX, USA). Odds ratios (ORs) with their 95% confidence intervals (95%CIs) were calculated as estimates of relative risk for CVD in ESRD patients. The *Z* test was used to estimate the statistical significance of pooled ORs. Heterogeneity among studies was estimated by the Cochran's *Q*-statistic and *I^2^* tests [26]. If a *Q*-test shows a *P*<0.05 or *I^2^*>50%, indicating significant heterogeneity, the random-effects model was conducted; otherwise the fixed-effects model was used. We also explored reasons for heterogeneity using meta-regression and subgroup analyses. In order to evaluate the influence of single studies on the overall estimate, a sensitivity analysis was performed. Funnel plots and Egger's linear regression test were applied to investigate publication bias [27].

## Results

### Eligible studies

**Figure 1:**
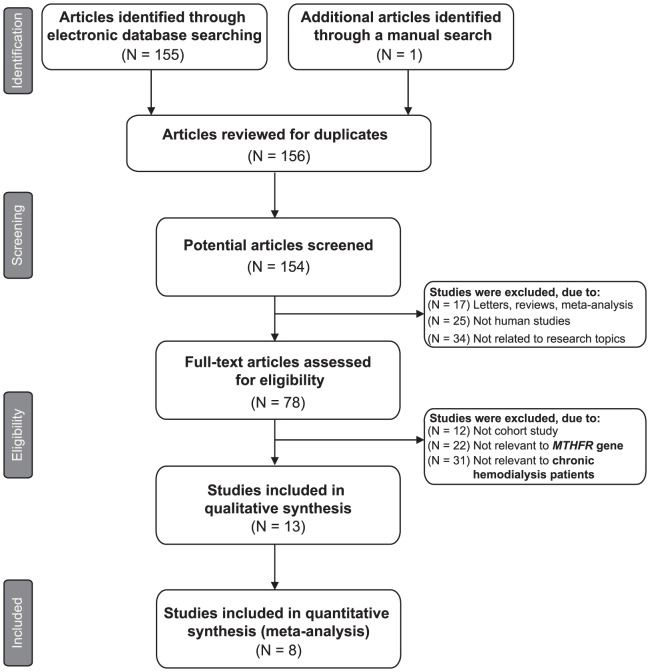
Flow chart shows study selection procedure. Eight cohort studies were included in this meta-analysis.

**Figure 2:**
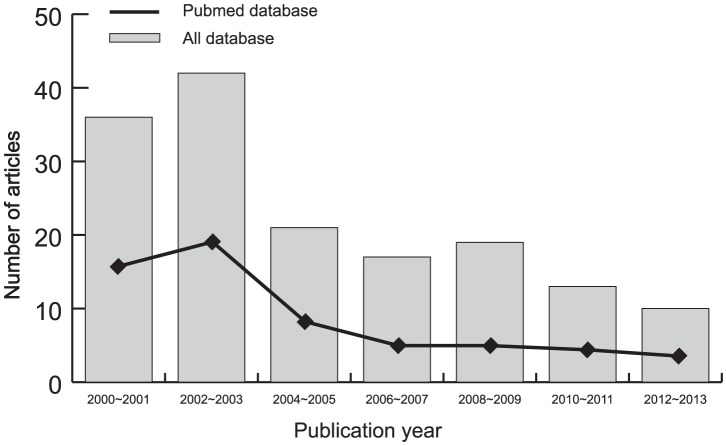
The distribution of the number of topic-related literatures in the electronic database over the last decade.

Initially, the highly sensitive search strategy identified 156 articles. After screening the titles and abstracts of all retrieved articles, 78 articles were excluded. Then full texts were reviewed and 65 articles were further excluded. Another 5 studies were also excluded due to lack of data integrity (Figure 1). Finally, 8 cohort studies were included in this meta-analysis [21], [23], [24], [28]–[32]. Publication years of the eligible studies ranged from 2000 to 2011. Figure 2 shows the distribution of the number of topic-related literatures in electronic databases over the last decade. A total of 2,292 ESRD patients with CVD were involved in this meta-analysis. Four studies were conducted among Asians, three studies among Caucasians, and one study among Africans. The polymerase chain reaction-amplified genes with restriction endonucleases (PCR-RFLP) was performed in 5 studies, while 3 studies used the direct DNA sequencing method. The genotype frequencies of controls were all in HWE (all *P*>0.05). The NOS scores of all included studies were ≥6. The main characteristics of all eligible studies are listed in Table 1.

**Table 1:** Baseline characteristics and methodological qualities of all included studies.

First author	Year	Country	Ethnicity	Case number	Gender (M/F)	Age (years)	Genotyping method	Genotype frequency			HWE test	NOS score
								CC	CT	TT	(*P* value)	
Lee et al [21]	2011	Korea	Asians	152	83/69	56.8±13.6	PCR-RFLP	44	72	36	0.538	7
Ibrahim et al [30]	2009	Egypt	Africans	50	29/21	41.6±11.8	Direct sequencing	30	16	4	0.385	6
Aucella et al [28]	2005	Italy	Caucasians	461	217/244	58.8±15.6	PCR-RFLP	134	220	86	0.801	8
Fukasawa et al [23]	2003	Japan	Asians	337	196/141	61.5±13.7	Direct sequencing	111	158	68	0.390	8
Morimoto et al [32]	2002	Japan	Asians	168	87/81	60.7±13.1	PCR-RFLP	78	67	23	0.167	7
Haviv et al [29]	2002	USA	Caucasians	120	72/48	48.0±13.0	PCR-RFLP	40	60	19	0.656	7
Wrone et al [24]	2001	USA	Caucasians	459	230/226	-	Direct sequencing	216	184	59	0.049	7
Kimura et al [31]	2000	Japan	Asians	545	319/226	58.7±12.5	PCR-RFLP	199	251	95	0.303	8

Legend: M – male; F – female; SNP - single nucleotide polymorphism; HWE - Hardy-Weinberg equilibrium; NOS - Newcastle-Ottawa Scale; PCR-RFLP - polymerase chain reaction-restriction fragment length polymorphism.

### Quantitative data synthesis

**Figure 3:**
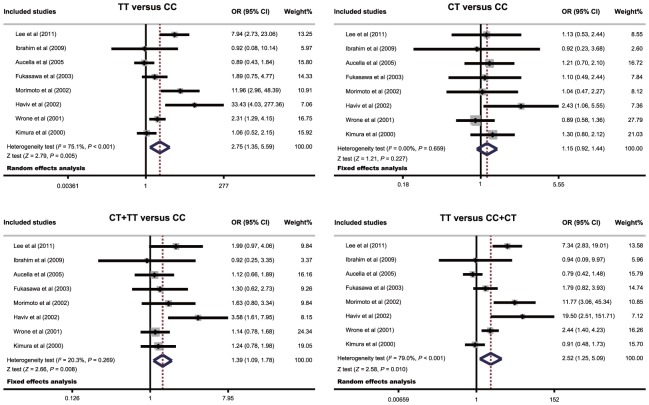
Forest plots for the relationships between the *MTHFR* 677C>T polymorphism and the risk of cardiovascular disease in chronic hemodialysis patients.

Meta-analysis findings on the relationship between the *MTHFR* 677C>T polymorphism and susceptibility to CVD in ESRD patients is shown in Table 2. The random-effects model was conducted due to significant heterogeneity between studies. Our meta-analysis results revealed that the *MTHFR* 677C>T polymorphism might increase the risk of CVD in ESRD patients (TT vs. CC: OR = 2.75, 95%CI = 1.35∼5.59, *P* = 0.005; CT+TT vs. CC: OR = 1.39, 95%CI = 1.09∼1.78, *P* = 0.008; TT vs. CC+CT: OR = 2.52, 95%CI = 1.25∼5.09, *P* = 0.010; respectively) (Figure 3).

**Table 2:** Meta-analysis of the relationships between the *MTHFR* 677 C>T polymorphism and the risk of cardiovascular disease in patients with end-stage renal disease.

	Sample size	TT vs. CC			CT vs. CC			CT+TT vs. CC			TT vs. CC+CT		
		OR	95%CI	*P*	OR	95%CI	*P*	OR	95%CI	*P*	OR	95%CI	*P*
Overall		2.75	1.35–5.59	0.005	1.15	0.92–1.44	0.227	1.39	1.09–1.78	0.008	2.52	1.25–5.09	0.010
*Ethnicity*													
Asians (n = 4)	1202	3.38	1.11–10.28	0.032	1.18	0.85–1.65	0.327	1.44	1.05–1.97	0.022	3.15	1.02–9.72	0.046
Africans (n = 1)	50	0.92	0.08–10.14	0.943	0.92	0.23–3.68	0.902	0.92	0.25–3.35	0.895	0.94	0.09–9.97	0.962
Caucasians (n = 3)	1040	2.72	0.76–9.78	0.125	1.25	0.75–2.07	0.389	1.52	0.84–2.73	0.165	2.35	0.69–7.96	0.171
*Genotyping method*													
PCR-RFLP (n = 5)	1446	4.00	1.19–13.53	0.025	1.31	0.98–1.74	0.065	1.61	1.11–2.32	0.012	3.42	1.04–11.23	0.043
Direct sequencing (n = 3)	846	2.11	1.30–3.42	0.003	0.93	0.65–1.34	0.695	1.16	0.83–1.61	0.394	2.14	1.37–3.33	0.001

Legend: OR - odds ratio; 95%CI - 95% confidence interval; PCR-RFLP - polymerase chain reaction-restriction fragment length polymorphism.

Subgroup and meta-regression analysis were conducted based on ethnicity to investigate potential sources of heterogeneity. Our results suggested that the *MTHFR* 677C>T polymorphism was associated with the incidence of CVD in ESRD patients among Asians (TT vs. CC: OR = 3.38, 95%CI = 1.11∼10.28, *P* = 0.032; CT+TT vs. CC: OR = 1.44, 95%CI = 1.05∼1.97, *P* = 0.022; TT vs. CC+CT: OR = 3.15, 95%CI = 1.02∼9.72, *P* = 0.046; respectively), but not among Africans or Caucasians (all *P*>0.05). Further subgroup analyses based on genotyping method suggested that the *MTHFR* 677C>T polymorphism was closely linked to an increased risk of CVD in the majority of subgroups (as shown in Table 2).

**Figure 4:**
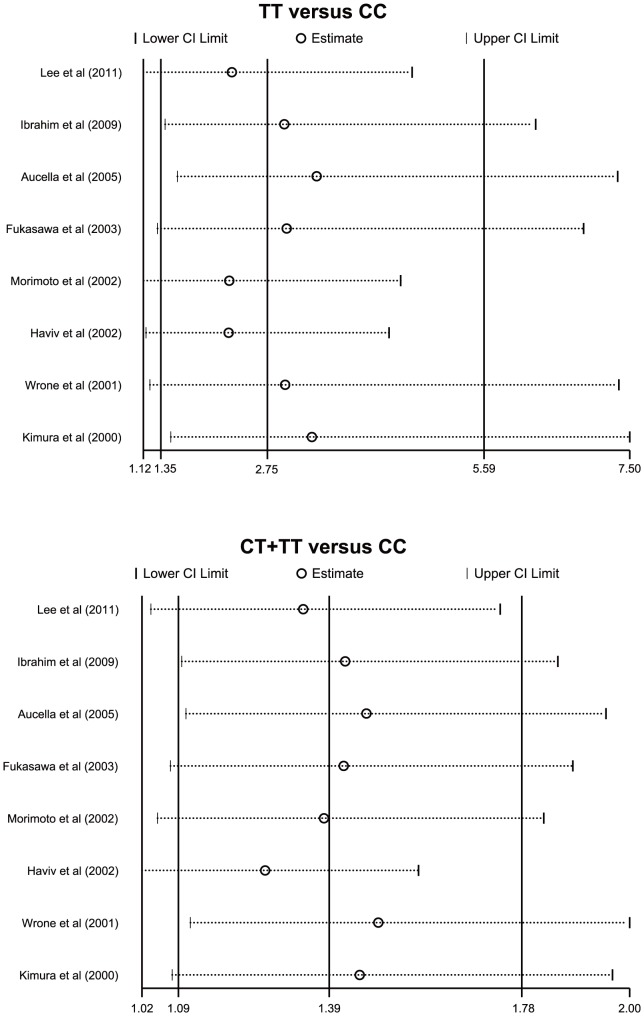
Sensitivity analysis of the summary odds ratio coefficients on the relationships between the *MTHFR* 677C>T polymorphism and the risk of cardiovascular disease in chronic hemodialysis patients.

**Figure 5:**
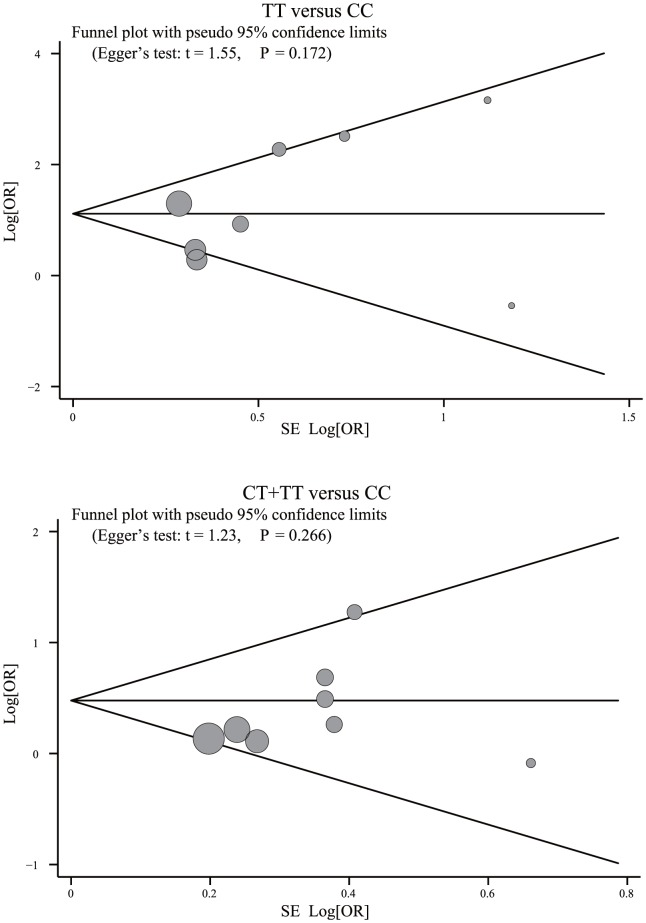
Funnel plot of publication biases on the relationships between the *MTHFR* 677C>T polymorphism and the risk of cardiovascular disease in chronic hemodialysis patients.

Meta-regression analysis confirmed that none of the considered factors was the main source of heterogeneity (as shown in Table 3). Sensitivity analysis suggested that no single study could influence the overall pooled estimates (Figure 4). We found no evidence of obvious asymmetry in the funnel plots (Figure 5). Egger's test also did not display strong statistical evidence for publication bias (all *P*>0.05).

**Table 3:** Univariate and multivariate meta-regression analyses of potential source of heterogeneity.

Heterogeneity factors	Coefficient	SE	*Z*	*P*	95%CI	
					LL	UL
*Publication year*						
Univariate	0.028	0.122	0.23	0.820	−0.212	0.267
Multivariate	−0.078	0.067	−1.17	0.241	−0.209	0.053
*Ethnicity*						
Univariate	−0.439	0.697	−0.63	0.528	−1.806	0.927
Multivariate	−0.132	0.339	−0.39	0.697	−0.796	0.532
*Genotyping method*						
Univariate	−0.769	0.922	−0.83	0.405	−2.577	1.039
Multivariate	0.562	0.372	1.51	0.131	−0.167	1.291

Legend: SE - standard error; 95%CI - 95% confidence interval; UL - upper limit; LL - lower limit.

## Discussion

MTHFR can catalyze a rate-limiting reaction in the transformation of methionine into Hcy and back exclusively through the transfer of the methyl group to and from methyl-tetrahydrofolate, separately [33]–[35]. It is well documented that an elevated level of the sulphur amino-acid Hcy in plasma is an independent risk factor for atherosclerosis, coronary heart disease, stroke and other CVDs [36], [37]. Meanwhile, the increased fasting level of total Hcy was found to establish an independent contribution to the excess incidence of fatal and non-fatal cardiovascular outcomes [38]. Indeed, the formation of hyperhomocysteinemia (HHcy) was closely associated with folic acid, Vitamin B_6_ and B_12_ supplementation [39], but also correlated with sulfur amino acid metabolism [40]. As for *MTHFR*, since the methyl groups for Hcy mediated DNA-methylation to methionine are related to the provision of 5-methylenetetrahydrofolate, which can be irreversibly transferred from 5 to 10-methylenetetrahydrofolate through MTHFR catalysis, the *MTHFR* gene probably plays a crucial role in the re-methylation of Hcy [41]. On the other hand, it has been estimated by statistical data in clinical studies that HD patients suffering from mild to moderate HHcy account for a great proportion of the high-risk population for CVD [42]. Therefore, it was hypothesized that common polymorphisms in the *MTHFR* gene could be functional and associated with the development and progression of CVD in ESRD patients. Recently, the usage of human *MTHFR* gene mutations in predicting the increased Hcy levels and susceptibility to CVD has been widely adopted [13], [34]. In particular, a relatively common functional polymorphism in *MTHFR* rs1801133 (677C>T) in exon 4 may influence the plasma Hcy in ESRD patients. Abundant evidence has supported the view that a half reduction in enzymatic capability might lead to a mild Hcy, as well as total elevation of plasma Hcy concentrations in individuals, both of which might contribute to the genetic predisposition to CVD [30], [43].

We undertook the present meta-analysis to elucidate the association of genetic mutations in the *MTHFR* gene with the incidence of CVD in ESRD patients. Our meta-analysis results showed that the *MTHFR* 677C>T polymorphism was associated with an increased risk of CVD in ESRD patients, indicating that variants in the *MTHFR* gene may play a pivotal role in the pathology of CVD in ESRD patients. Although the mechanism by which *MTHFR* genetic polymorphisms lead to the development of CVD in ESRD patients still remains partially understood, a possible explanation is that the *MTHFR* 677C>T polymorphism may lead to a mild decrease in MTHFR activity which plays a crucial role in the remethylation of Hcy to methionine, resulting in elevated total plasma Hcy levels which may contribute independently to the excess incidence of fatal and nonfatal CVD. Haviv YS et al. have suggested a major role for plasma Hcy level as a determinant of CVD in ESRD patients, whereby the *MTHFR* variants and low serum folate are important determinants of plasma Hcy level and therefore may also have an indirect impact on the cardiovascular status [29]. Since heterogeneity obviously existed in our meta-analysis, we performed stratified analyses based on ethnicity and genotyping method. Subgroup analysis by ethnicity indicated that there were marked associations between the *MTHFR* 677C>T polymorphism and an increased risk of CVD in ESRD patients among Asians. In short, our findings were in accordance with previous studies that found that the *MTHFR* 677C>T polymorphism may alter the risk of developing CVD in ESRD patients, suggesting that these polymorphisms may be useful as biomarkers for the early diagnosis of CVD in ESRD patients.

As the first meta-analysis focused on the relationships between the *MTHFR* 677C>T polymorphism and the risk of CVD in ESRD patients, our study has some limitations. First, our results lacked sufficient statistical power to assess correlations between the *MTHFR* 677C>T polymorphism and susceptibility to CVD in ESRD patients due to a relatively small sample size. Second, as a retrospective study, our meta-analysis may have been influenced by subject selection bias, thereby affecting the reliability of our results. Third, our meta-analysis failed to obtain original data from the included studies, which may have limited further evaluation of the potential roles of the *MTHFR* 677C>T polymorphism in the development and progression of CVD in ESRD patients. Importantly, the inclusion criteria of cases and controls were not well defined in all included studies, which might also have influenced our results.

In conclusion, our findings indicate that the *MTHFR* 677C>T polymorphism may contribute to increased CVD risk in ESRD patients. Thus, the *MTHFR* 677C>T polymorphism may be a useful and promising biomarker for the early detection and prognosis of CVD in ESRD patients. However, due to the limitations mentioned above, more research studies with larger sample sizes are still needed to provide a more representative statistical analysis.

## Supporting Information

Checklist S1 **The PRISMA 2009 checklist.**
(DOC)

Supplement S1 **The Newcastle-Ottawa Scale for assessing methodological quality.**
(DOC)
