# Correction: Correlations of *MTHFR* 677C>T Polymorphism with Cardiovascular Disease in Patients with End-Stage Renal Disease: A Meta-Analysis

**DOI:** 10.1371/journal.pone.0116443

**Published:** 2014-12-19

**Authors:** The PLOS ONE Staff

The following information is missing from the Funding section: This work was financially supported by the Department of Education of Liaoning province (L20120113).
